# Functional EL-HN Fragment as a Potent Candidate Vaccine for the Prevention of Botulinum Neurotoxin Serotype E

**DOI:** 10.3390/toxins14020135

**Published:** 2022-02-11

**Authors:** Zhen Li, Jiansheng Lu, Xiao Tan, Rong Wang, Qing Xu, Yunzhou Yu, Zhixin Yang

**Affiliations:** 1 Beijing Institute of Biotechnology, Beijing 100071, China; lz08978@163.com (Z.L.); lujiansheng2008@163.com (J.L.); 20121612@bjtu.edu.cn (X.T.); wangrong_8312@163.com (R.W.); 2 Institute of Life Science and Biotechnology, Beijing Jiaotong University, Beijing 100044, China

**Keywords:** botulinum neurotoxin, functional fragment, L-HN fragment, subunit vaccine, immunoprotective effect

## Abstract

*Clostridium botulinum* produces botulinum neurotoxin (BoNT), which is the most toxic known protein and the causative agent of human botulism. BoNTs have similar structures and functions, comprising three functional domains: catalytic domain (L), translocation domain (HN), and receptor-binding domain (Hc). In the present study, BoNT/E was selected as a model toxin to further explore the immunological significance of each domain. The EL-HN fragment (L and HN domains of BoNT/E) retained the enzymatic activity without in vivo neurotoxicity. Extensive investigations showed EL-HN functional fragment had the highest protective efficacy and contained some functional neutralizing epitopes. Further experiments demonstrated the EL-HN provided a superior protective effect compared with the EHc or EHc and EL-HN combination. Thus, the EL-HN played an important role in immune protection against BoNT/E and could provide an excellent platform for the design of botulinum vaccines and neutralizing antibodies. The EL-HN has the potential to replace EHc or toxoid as the optimal immunogen for the botulinum vaccine.

## 1. Introduction

Botulinum neurotoxin (BoNT) is a neurotoxin produced by *Clostridium botulinum* in an anaerobic environment [1,2]. BoNT is the most toxic protein among bacteria, animals, plants, and chemical substances reported to date. BoNTs are classified immunologically into distinct serotypes: from BoNT/A to BoNT/G. Botulinum neurotoxin serotypes A, B, E, and F cause botulism in humans. Botulinum neurotoxin has been classified as an A-class biological warfare agent and an important bioterrorism agent because of its high toxicity and relative ease of production and transportation [3,4]. Novel types of BoNTs (e.g., BoNT/HA and BoNT/X) have also been identified in recent years [1,5].

BoNT/A–G have similar structures and functions, with amino acid sequence homology of 37–70% [6]. BoNT consists of two chains (L and H) connected by an S–S bridge. BoNTs have an approximate molecular mass of 150 kDa and comprise three major functional domains. The L-chain (L, 50 kDa) contains the catalytic domain, the C-terminal part of the H-chain is the receptor-binding domain (Hc, 50 kDa), and the N-terminal part is the translocation domain (HN, 50-kDa). The Hc domain is involved in the binding of neuronal cells [7,8,9]. The heavy chain N-terminal transmembrane transport functional domain (HN), which plays an important role in the transmembrane transport of toxins, is necessary for toxins to enter into cells [10]. The L domain, a fold composed of an α-helix and β-sheet bundle, has zinc endopeptidase activity [11,12,13].

The intoxication process is divided into three steps. First, the Hc domain of BoNT binds to the neuronal membrane on the presynaptic side of the peripheral synapse [9]. Then, this binding triggers the translocation of BoNT into the neuronal cytosol by receptor-mediated endocytosis. The light chain of the toxin passes through the endocytic vesicle membrane and enters the cytoplasm. Upon entry into the cytoplasm, the light chain cleaves polypeptides that are essential for neurotransmitter release. Thereby, the extracellular release of neurotransmitters into the neuromuscular junction is blocked, resulting in paralysis of the airways and respiratory muscles that can lead to ventilator failure and death [2].

Vaccination is an effective strategy to prevent botulism. An investigational pentavalent vaccine (Pentavalent Botulinum Toxoid Vaccine, PBT) has been the most used human botulism vaccine, which induces neutralizing antibodies against BoNT serotypes A, B, C, D, and E. However, the availability of this vaccine is limited because it is expensive, difficult to produce, and involves a dangerous detoxification process [14]. Therefore, there is an urgent need to develop an efficient, safe, and convenient vaccine for the prevention of botulism. A novel vaccine-design strategy is developed using the nontoxic recombinant domains of BoNTs as antigens, but not a full toxin. Present research on BoNT vaccines and neutralizing antibodies have primarily used Hc as the target antigen [15]. A bivalent recombinant Hc subunit vaccine rBV A/B for the prevention of BoNT/A and BoNT/B was developed and is undergoing phase II clinical trials [16].

Among the various serotypes of BoNTs, serotype A has attracted the most attention from researchers in various countries, while fewer studies have investigated serotype E. In terms of molecular structure, the L and Hc domains of BoNT/E are located on the same side of the HN domain. It has a unique domain arrangement, which was suggested to be linked to the faster onset of action, making the symptoms of poisoning appear more rapidly, with an impact on human health that cannot be underestimated [17]. Botulism of serotype E occurs frequently, mostly in coastal areas, and it is strongly associated with the consumption of contaminated marine life and other fishery products [18]. Therefore, the immunological characterization of toxin molecules is important to design efficient vaccines and antibodies to prevent and treat botulism of BoNT/E.

In the present study, BoNT/E was selected as a model toxin to further explore the immunological significance of each domain. We prepared a series of recombinant antigens derived from the different domains of BoNT/E and systematically explored their immunological characteristics and immunoprotective efficacies. Our results showed that the recombinant EL-HN fragment (L and HN domains of BoNT/E), which was described previously by Shone et al. [19], has native neurotoxin activity and could generate powerful neutralizing antibodies, playing an important role in immune protection against BoNT/E. Therefore, it could be used as a recombinant subunit vaccine against BoNT/E.

## 2. Results

### 2.1. Purification and Analysis of Recombinant Functional Fragment Antigens

**Figure 1:**
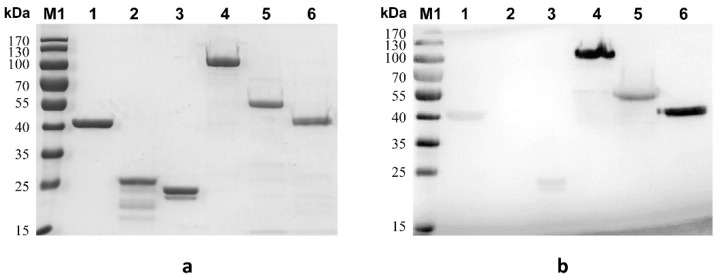
Identification of recombinant fragment antigens by SDS-PAGE (**a**) and Western blotting (**b**). Lane 1, EHc; lane 2, EHc-N; lane 3, EHc-C; lane 4, EL-HN; lane 5, EHN; lane 6, EL; (**b**) Western blotting analysis utilizing antibodies from hyperimmune anti-BoNT/E horse sera.

Table 1 shows the details of the BoNT/E-derived functional fragment antigens produced in the present study. These recombinant functional fragments were expressed in *E. coli* (BL21), and the purified proteins were identified by both their molecular weight and reaction with specific antibodies to BoNT/E in immunoblots. SDS-PAGE analysis (Figure 1a) showed that recombinant antigens were obtained after purification using Ni-NTA affinity chromatography. The sera antibodies against BoNT/E could bind to the recombinant antigens EHc, EHc-C, EL-HN, EHN, and EL as assessed using Western blotting (Figure 1b), which indicated that these recombinant proteins have antigenicity to BoNT/E. Our results confirmed these recombinant antigens were from fragments of BoNT/E.

### 2.2. Function and Property of the Recombinant EL-HN Fragment

**Figure 2:**
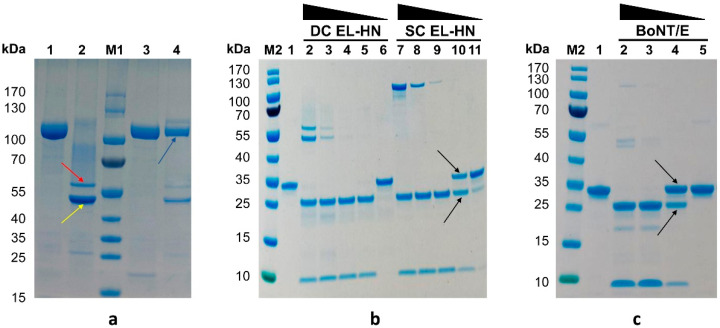
Function and property of EL-HN fragment derived from the L and HN domains of BoNT/E. (**a**) Analysis of the EL-HN following nicking by trypsin. The un-nicked EL-HN fragment (single chain, SC) and nicked EL-HN fragment (dichain, DC) were determined by SDS-PAGE in the presence or absence of reductant. Lanes 1 and 3, un-nicked EL-HN fragment (100 kDa); lanes 2 and 4, nicked EL-HN fragment. The red arrow indicates the position of the EHN fragment. The yellow arrow indicates the position of the EL fragment. Blue arrows indicate the position of the EL-HN fragment. (**b**,**c**) Analysis of substrate SNAP-25 following cleaved by the EL-HN fragments and BoNT/E. (**b**) Catalytic activity of un-nicked or nicked EL-HN (lanes 2–6 with DC EL-HN; lanes 7–11 with SC EL-HN); lane 1, 2 μmol SNAP-25; lanes 2 and 7, 2 μmol SNAP-25 + 1600 nmol EL-HN; lanes 3 and 8, 2 μmol SNAP-25 + 320 nmol EL-HN; lanes 4 and 9, 2 μmol SNAP-25 + 64 nmol EL-HN; lanes 5 and 10, 2 μmol SNAP-25 + 12.8 nmol EL-HN; lanes 6 and 11, 2 μmol SNAP-25 + 2.56 nmol EL-HN; (**c**) Catalytic activity of BoNT/E. Lane 1, 5 μmol SNAP-25; lane 2, 5 μmol SNAP-25 + 600 nmol BoNT/E; lane 3, 5 μmol SNAP-25 + 120 nmol BoNT/E; lane 4, 5 μmol SNAP-25 + 24 nmol BoNT/E; lane 5, 5 μmol SNAP-25 + 4.8 nmol BoNT/E. Arrows indicate uncleaved and cleaved SNAP-25.

The SDS-PAGE results (Figure 2a) showed that the un-nicked EL-HN fragment yielded a single 100 kDa band upon SDS-PAGE in either the presence or absence of reductant, confirming that it is expressed in the SC form. Controlled nicking with trypsin resulted in a complete conversion of the single chain (SC) to a disulfide-linked dichain (DC) as revealed by the appearance of the EL and EHN via SDS-PAGE in the presence of the reductant. The nicked EL-HN in the absence of reductant was revealed as a disulfide-linked DC (100 kDa).

The enzymatic activity of EL-HN fragments to cleave efficiently their substrate SNAP-25 is explored in this study. Incubation of SNAP-25 with EL-HN resulted in cleavage of SNAP-25 and produced two small fragments (Figure 2b). The percentage cleavage of the substrate by different concentrations of the enzyme showed that 12.8 nmol DC EL-HN was able to completely cleave 2 μmol SNAP-25 in 30 min of incubation at 37C, while 64 nmol SC EL-HN completely cleaved 2 μmol SNAP-25. The nicked EL-HN (DC) has stronger catalytic activity than the un-nicked EL-HN (SC). Our results suggest that the EL-HN functional fragment has a similar level of catalytic activity to BoNT/E (Figure 2c).

### 2.3. Specific Neurotoxicity of the Recombinant EL-HN Fragment

The neurotoxicity of the un-nicked and nicked EL-HN fragment was determined following intraperitoneal injection into mice. There were no obvious toxic symptoms in the mice injected with 50 μg of the un-nicked EL-HN, whereas low neurotoxicity was observed in mice injected with the nicked EL-HN, which may owe to the stronger catalytically activity or DC property. The LD_50_ observed for the nicked EL-HN was 0.5 μg, which equates to 2 × 10^3^ LD_50_/mg of protein. The toxicity of BoNT/E lacking the Hc domain was found to be greatly reduced compared to the wild-type toxin. Therefore, the EL-HN fragment as an antigen was considered to be safe.

### 2.4. Immunoprotective Efficacy of the Functional Fragment Antigens

In all immunological experiments, the un-nicked EL-HN (single chain, SC) was used to immunize mice. The survival rate of toxin challenge and the level of neutralization titer were used to evaluate the immunoprotective potency of the recombinant antigens. As demonstrated in Table 2, good immunoprotective effects were observed for both EHc and EL-HN antigens produced in mice after two immunizations and could completely protect mice against challenges by 10^4^ LD_50_ BoNT/E. Very high levels of serum neutralization antibodies were observed in these immunized mice. Two or three immunizations with EHc-C, EHN, and EL produced weak immune protection in mice (*p* < 0.05, compared with both EHc and EL-HN) with low levels of serum neutralization antibodies. After the third immunization, the protective potency of the above antigens was moderately enhanced. However, the EHc-N antigen did not produce immune protection in mice after three immunizations, and the serum neutralization antibody titer was less than the low measured value of 0.25 IU/mL.

The neutralizing antibody response produced by the combination antigens of EHc-N + EHc-C was also lower than that produced by EHc alone (Table 2). We speculated that the structural integrity of EHc was critical to the protective effect. Moreover, the protective potency of the EL-HN fragment was stronger than that of EL and EHN alone or the EL and EHN combination, indicating that there may be important neutralizing antibody epitopes at the junction of domain EL and EHN.

### 2.5. Determination of the Humoral Immune Response after Immunization with Different Functional Fragment Antigens

**Figure 3:**
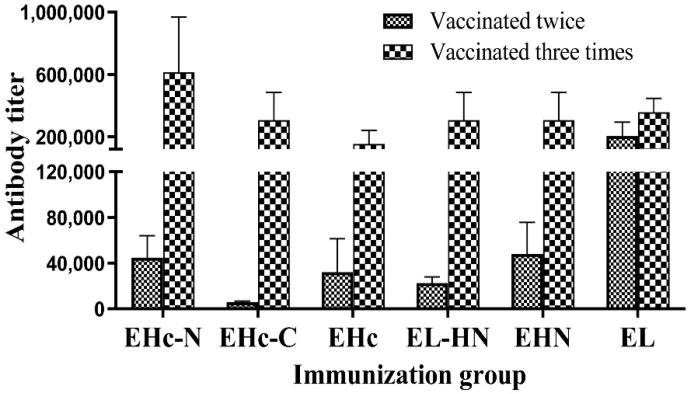
Serum antibody titer in mice after immunization using different functional fragment antigens. Serum samples of the individual mice (*n* = 10, each group) were obtained after two or three immunizations, and ELISA was used to measure the specific antibody titer. Individual mouse serum samples were assayed, and for each group, the geometric mean titer (GMT) was calculated.

ELISA was used to detect antibody titer in mouse sera after immunization with the recombinant antigens. Each recombinant antigen induced a strong and specific antibody reaction against itself, and the antibody titer after the three vaccinations were significantly enhanced (Figure 3). These results indicated that the recombinant antigens had good immunogenicity and induced a strong antibody response.

**Figure 4:**
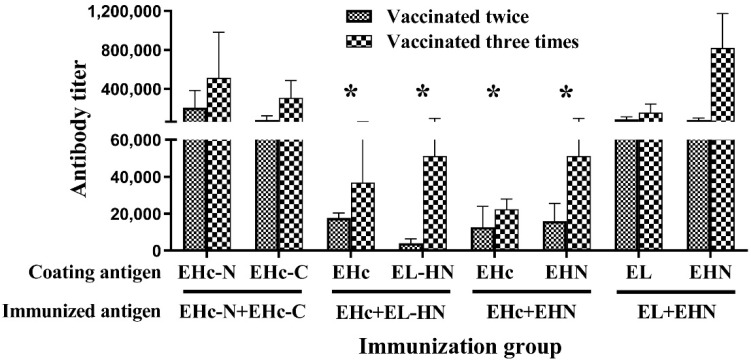
Serum antibody titer in mice after immunization using combination of two fragment antigens. Serum samples of the individual mice (*n* = 10, each group) were obtained after two or three immunizations, and ELISA was used to measure the specific antibody titer. Individual mouse serum samples were assayed, and for each group, the geometric mean titer (GMT) was calculated. * *p* < 0.05; anti-EHc, EHN, or EL-HN antibody titer of combined groups (EHc + EHN or EHc + EL-HN) were compared with that of the single antigen.

The antibody titer in mice induced by the combined antigen were detected using plates coated with the single component of the combined antigen. According to the results, the antibody response induced by EHc + EHN or EHc + EL-HN was weaker than that of a single antigen (Figure 3 and Figure 4, *p* < 0.05), which might be related to the structure of the antigen and the immunity of the combined antigen. It also led to a protective effect that was lower than that of the single antigen.

### 2.6. Dose-Dependent Protective Potency of the Recombinant Antigens

**Figure 5:**
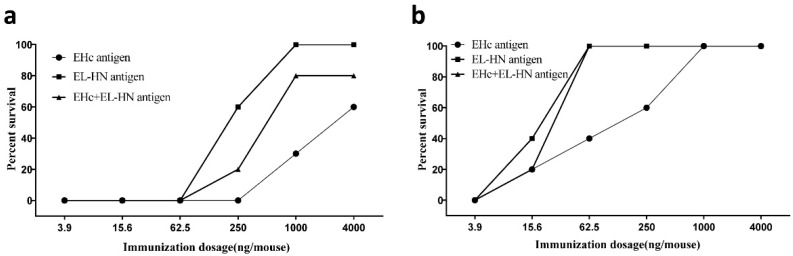
Rate of survival among mice immunized using different dosages of EHc or/and EL-HN antigens. (**a**) BoNT/E at 10^2^ LD_50_ was used to challenge mice 3 weeks after the first immunization. (**b**) Mice were challenged with 10^3^ LD_50_ of BoNT/E 3 weeks after two immunizations.

To study the protective potency of certain recombinant antigens, dose-dependent protective tests were carried out for EHc, EL-HN, and EHc + EL-HN, which had better immunoprotective effects. The mice immunized once with different dosages of recombinant antigens EHc, EL-HN, and EHc + EL-HN were challenged with 10^2^ LD_50_ of BoNT/E. The survival rate of the EL-HN group with 4 μg or 1 μg was 100%. The survival rate of the EHc + EL-HN group with 4 μg or 1 μg was 80%, and that of the EHc group with 4 μg or 1 μg was 60% or 30% (Table 3). The ED_50_ values for immunization with one dose of EHc, EL-HN, and EHc + EL-HN were 2.557 μg, 0.223 μg, and 0.703 μg, respectively (Figure 5a).

The mice immunized twice with different dosages of recombinant antigens EHc, EL-HN, and EHc + EL-HN were challenged with 10^3^ LD_50_ of BoNT/E. The survival rate of the EL-HN and EHc + EL-HN groups with two injections of ≥62.5 ng dosage was 100%. The survival rate of the EHc group with the 62.5 ng dosage was 40% (*p* = 0.011< 0.05). Only immunization with ≥1 μg of EHc could provide 100% protection (Table 3). The ED_50_ values for immunization with two doses of EHc, EL-HN, and EHc + EL-HN were 0.092 μg, 0.017 μg, and 0.023 μg, respectively (Figure 5b). Thus, the dose-dependency experiments showed that the immunogen with the highest protection efficacy against BoNT/E was the EL-HN antigen.

**Figure 6:**
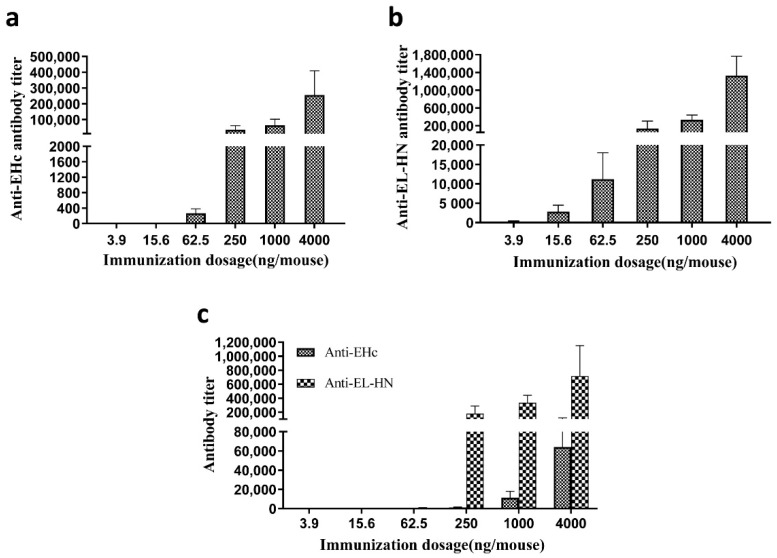
Determination of antibody titer in mice that received two immunizations with different amounts of EHc or/and EL-HN antigens. (**a**) Anti-EHc antibody titer in mice immunized with EHc antigen. (**b**) Anti-EL-HN antibody titer in mice immunized with EL-HN antigen. (**c**) Anti-EHc or EL-HN antibody titer in mice immunized with EHc + EL-HN antigen. Serum samples of the individual mice (*n* = 10, each group) were obtained after two immunizations, and ELISA was used to measure the specific antibody titer. Individual mouse serum samples were assayed, and for each group, the geometric mean titer (GMT) was calculated.

Furthermore, the antibody titer in mouse sera after two immunizations with different dosages of recombinant antigens EHc and/or EL-HN were determined (Figure 6). The levels of anti-EHc or EL-HN and neutralizing antibodies were elicited in a dose-dependent manner and correlated positively with their protective effects (Table 3 and Figure 4). Mice receiving two immunizations of the EL-HN antigen (≥62.5 ng) had high titer of serum neutralizing antibodies (≥1 IU/mL) and could protect against BoNT/E at 10^3^ LD_50_. Meanwhile, two immunizations of the EHc antigen at ≥1 μg yielded relatively high titer of serum neutralizing antibodies (≥0.5 IU/mL). In summary, two immunizations with the EL-HN antigen at a low dose provided complete protection for mice challenged with a high dose of BoNT/E.

## 3. Discussion

BoNT has been classified as an important biological threat because of its high toxicity and lethality [1]. Botulism could be prevented effectively via vaccination. In the last 20 years, the immunological significance of the Hc domain of BoNTs has been the subject of intensive research, leading to important findings [14,15,16,20,21,22,23,24,25]. Our research also indicated that the Hc domain is an effective protective antigen in subunit vaccines for BoNT/A, B, E, and F [26,27,28,29]. However, the immunogenicity of other regions requires further study, such as their protective efficacy and the synergistic effects of multiple epitopes. The L and HN region of BoNTs has been explored as a vaccine candidate, indicating that the L and/or HN domains are valuable regions to generate protective antibodies [30,31,32,33,34].

As another novel research direction, chimeric or fusion molecules with two domains of BoNTs have promising research value [14]. Protection against BoNTs was achieved using a bivalent recombinant vaccine for BoNT/A and B based on their translocation and effector domains [35]. Recently, we explored the immunological characterization and immunoprotective efficacy of BoNT/A functional fragment antigens [36]. Our results showed that a recombinant *E.coli*-expressed AL-HN protein without formaldehyde treatment displayed a stable structure and antigenicity. This AL-HN antigen, containing important neutralizing epitopes, produced immune protection, although its protective potency was weaker than that of AHc. Moreover, we found that another antigen BL-HN derived from BoNT/B could provide effective protection; however, its protective potency was also weaker than that of BHc [37].

In the present study, we explored the immunological characterizations and immunoprotective efficacy of different functional fragments derived from BoNT/E. Notably, we found that the protective potency of EL-HN was significantly stronger than that of EHc or the other fragments, which was contrasted with the results for these L-HN fragments of BoNT/A and B. Immunization of EL-HN induced very high levels of serum neutralizing antibodies in mice and two immunizations with a low dosage of the EL-HN antigen can induce high levels of neutralizing antibodies. In addition, the protective effect of the EL + EHN combination antigen was stronger than that of EL or EHN alone, indicating that the combination of the two domains had a certain synergistic effect. However, the protective potency of the combination is lower than this EL-HN fragment. Therefore, our data indicated that the EL and EHN domains contain functional neutralizing epitopes and the functional EL-HN fragment is also an important protective antigen. More importantly, these results indicate that there are more effective neutralizing antibody epitopes on EL-HN, especially at the junction of EL and EHN, than on other fragments of BoNT/E.

Property and activity of EL-HN fragment were also defined in this study. Our results indicate that the *E.coli*-expressed EL-HN fragment may retain the enzymatic activity, which supports that functional neutralizing epitopes exist in the EL-HN fragment. The un-nicked EL-HN fragment was expressed as the sole SC protein and was observed to be in DC form after nicking by trypsin. The functional EL-HN fragment is catalytically active proteins and efficiently cleaves their substrate SNAP-25 as active BoNT/E.

In general, the BoNT antigenic epitopes are distributed widely among the full toxin, and the core immunogen for immunoprotection comprises the functional neutralizing epitopes. The L-HN fragment derived from BoNTs represents the N-terminal two-thirds of the neurotoxin. In the present study, *E. coli* was used to express the L-HN protein, which retains the functionality of the parent holotoxin but lacks the neuron-specific targeting contributed by the binding domains in the full-length BoNT, and retained its stable property, activity, and antigenicity. However, using BoNT/E as a model toxin, we found that the protective potency of EL-HN was significantly stronger than that of other fragment antigens, including EHc. These results are the first to reveal the strong protective effect of the L-HN fragment from BoNT/E.

Among the neutralizing antibodies against BoNT/E, many target the L [38] and HN [39] domains rather than the Hc domain [18,40,41]. In particular, Garcia-Rodriguez isolated single-chain antibody (scFv) libraries constructed from the VH and VK genes of healthy individuals immunized with pentavalent BoNT toxins (serotypes A, B, C, D, E, and F) [42]. These scFv strains were used to develop a monoclonal antibody-based antitoxin that can neutralize multiple BoNT/E subtypes. Yeast display was used to determine the binding position of ten monoclonal antibodies. One monoclonal antibody bound the Hc domain, two monoclonal antibodies bound non-overlapping HN epitopes, and six monoclonal antibodies bound the L domain. It was found that the tenth monoclonal antibody, 3E2, binds EL-HN but does not bind EHN and EL alone, indicating that there are neutralizing epitopes at the junction of EL and EHN. Further study found that 3E2 has the strongest neutralizing effect on BoNT/E compared with other monoclonal antibodies. A combination of these antibodies specific to the L and/or HN domain of BoNT/E also showed a synergistic and potent neutralization effect against BoNT/E. It is important to note that NTM-1633, used in preclinical or clinical investigations, is an equimolar combination of three monoclonal antibodies (3E2, 3E6.1, and 4E17.1) that bind and strongly neutralize BoNT/B. Both 3E6.1 and 4E17.1 bind to the HN domain of BoNT/B. These data indicate that neutralizing antibodies targeting the EL-HN fragment have strong neutralization activity and synergistic effects. These results are also consistent with the highest protective effect of the EL-HN functional fragment, which retains critical epitopes responsible for inducing strong neutralizing antibodies.

Generally, toxin-protective antibodies should have a neutralization capacity. The mechanism of action of neutralization antibodies is to inhibit the binding of the toxin to the neuronal cell surface receptor and clear the toxin in circulation [43,44]. In this regard, the Hc domain comprising the BoNT/E receptor binding domain is an important target, in which antibody binding to Hc subsequently prevents the receptor from binding to the toxin. To date, Hc-based vaccines and antibodies have been used to prevent and treat botulism [15,16,25,33]. Antibodies that bind to the L and HN regions are also capable of neutralizing the toxin [38,39,42]. The exact mechanisms by which they neutralize the toxin is unclear; however, these antibodies might prevent one or more of the following steps [30,33,45,46,47]: (1) inhibiting toxin transport across the cell membrane; (2) inhibiting toxin transport to its targets along nerve fibers; (3) blocking the toxin’s action on its target. It is likely that some of the neutralization antibodies, such as single-domain antibodies (nanobodies) targeting the L or/and HN domains, could enter into the cell to neutralize the toxin by endocytosis or using a chimeric toxin-based platform [48,49]. Therefore, the L or/and HN domains are also important antigens or epitope targets. The L-HN-based vaccines and antibodies might also be used to prevent and treat botulism mediated by BoNTs.

In this study, the serum antibodies of EL-HN-immunized mice had stronger neutralizing activity than those of EHc and other fragments, which indicated that EL-HN contains more or important functional neutralization epitopes that play an important role in immune protection against BoNT/E. Webb et al. [50] demonstrated that, despite the use of the two-vaccination method, Hc-based BoNT/E vaccines provided weak protection, with an ED_50_ of 223 ng. Furthermore, catalytically inactive *Clostridium botulinum* holoproteins (ciBoNT HPs) might provide a stronger immunological response that elicits relatively higher potency than the Hc-based BoNT/E vaccines. In the present study, EL-HN delivered via the two-vaccination approach resulted in an ED_50_ of 17 ng, which was similar to the potency of ciBoNT/E HP (ED_50_ = 12 ng). Therefore, the potency of EL-HN as a subunit vaccine is equivalent to holoproteins of BoNT/E. Przedpelski et al. [34] also demonstrated that M-BoNT/A1^W^ and LCHC_N_ are more potent vaccines than HC_C_. LCHC_N_ had a similar vaccine potency as M-BoNT/A1^W^. M-BoNT/A1^W^ elicited a common dominant antibody response to LCHC_N_, but a varied HC_C_ antibody response in mice. This may be a general concept applicable to other BoNTs. The full-length not toxic BoNTs or other derivations represent a novel strategy for the development of vaccines against botulism.

Our data strongly indicated that the EL-HN region is important to generate protective antibodies and should aid the design of BoNT/E vaccines. With regard to BoNT/E, EL-HN is more suitable as a subunit vaccine of BoNT/E than EHc or other antigens. In addition, it is interesting that the recombinant EL-HN functional fragment is used to screen for L-HN-specific, but not Hc-specific, neutralizing antibodies to treat botulism.

## 4. Conclusions

The functional EL-HN fragment had the highest protective efficacy and contained some functional neutralizing epitopes. Furthermore, the EL-HN retained the property and activity of the toxin. Thus, the EL-HN has the potential to replace EHc or toxoid as the optimal immunogen for the botulinum vaccine. The functional L-HN fragment provides an additional tool that allows us to understand the structure–function relationship of the different domains of BoNT in the process of intoxication or degree of immune protection. Further biological function and immunological characterizations of the L-HN fragment will be performed in future studies. These findings offer a new strategy to develop vaccines and therapeutic antibodies against BoNTs.

## 5. Materials and Methods

### 5.1. Animals and Ethics Statement

Female Balb/C and KM mice were obtained from Vital River Laboratory Animal Technology Co. Ltd. (Beijing, China). The mice were housed under pathogen-free conditions (Laboratory Animal Center, Academy of Military Medical Science, Beijing, China) and randomly assigned to different groups. All animal experiments were performed with the permission of the Institute of Animal Care and Use Committee (IACUC) at the Academy of Military Medical Science, and the ethical approval number was IACUC-DWZX-2019-017.

### 5.2. Production of Recombinant Functional Fragment Antigens Derived from BoNT/E

To produce recombinant functional fragment antigens of BoNT/E in *Escherichia coli*, gene fragments of each function fragment (EL, EHN, EL-HN, EHc, EHc-N, and EHc-C, Table 1) were ligated into a prokaryotic expression vector, pTIG-Trx, to produce recombinant expression plasmids. As previously reported [26,36], our laboratory constructed pTIG-Trx prokaryotic expression plasmids encoding the following BoNT/E molecule fragments with His-tag: EL (residues 1–422), EHN (residues 423–840), EL-HN (residues 1–840), EHc-N (residues 841–1063), EHc-C (residues 1056–1252), and EHc (residues 841–1252). The resulting recombinant plasmids were confirmed by sequencing.

Each recombinant plasmid was transformed into the *E. coli* expression strain, BL21 (DE3). A single colony was inoculated in a 2×YT medium containing 100 μg/mL ampicillin and cultured at 37 °C with shaking at 220 rpm until the absorbance at 600 nm (OD_600_) of 1.0 was reached. The cells were inoculated into 400 mL of 2×YT medium (containing ampicillin at 100 µg/mL) and cultured at 37 °C with shaking at 220 rpm until OD_600_ of 0.8 was reached. Then, isopropyl-β-D-thiogalactopyranoside (IPTG; Promega, Madison, WI, USA) was added to the cell culture such that its final concentration was 0.2~0.4 mM to induce the expression of recombinant antigens. The cells were cultured overnight at 16 °C and centrifuged at 6000× *g* for 30 min to collect the cells. The cells were then resuspended in a binding buffer (20 mM sodium phosphate, pH 7.4) and lysed using an ultrasonic homogenizer in an ice water bath. The cells were centrifuged at 12,000× *g* for 20 min at 4 °C to collect the supernatant.

An AKTA Explorer (GE Healthcare, Piscataway, NJ, USA) was used for chromatographic purification of these recombinant proteins with His-tag at the C-terminus from the supernatant. The filtered lysates were then loaded onto a HisTrap HP (GE Healthcare Bio-Sciences, Uppsala, Sweden) column that was pre-equilibrated with 10 column volumes (CV) of binding buffer. Gradient elution with elution buffer was used to elute the recombinant protein from the column. The eluted sample was collected, and the buffer of the final product was exchanged with phosphate-buffered saline (PBS, pH 7.4). The purity and specificity of the recombinant antigens were checked using sodium dodecyl sulfate polyacrylamide gel electrophoresis (SDS-PAGE) and Western blotting.

The antigenicity of the recombinant protein was detected by Western blotting. After separation by 10% SDS-PAGE, the EHc, EHc-N, EHc-C, EL-HN, EHN, and EL proteins were transferred to a polyvinylidene fluoride (PVDF) membrane using a film transfer apparatus and blocked with 5% skim milk at 37 °C for 1 h. The membrane was then incubated with 1:5000 diluted antibodies from hyperimmune anti-BoNT/E horse sera overnight at 4 °C. After washing with TBST (Tris-buffered saline with 0.1% Tween-20), the membrane was incubated with 1:2000 diluted goat anti-horse IgG-HRP (Santa Cruz Biotechnology Inc., Santa Cruz, CA, USA) at 37 °C for another 1 h. After washing with TBST, Western blotting solution (Thermo, Franklin, MA, USA) was added to the membrane, and it was exposed on a gel imager (Bio-Rad, Hercules, CA, USA).

### 5.3. Enzymatic Activity of EL-HN

The recombinant EL-HN fragment was proteolytically nicked by trypsin (NEB, Ipswich, MA, USA) (1:100 ratio of trypsin to EL-HN) for 30 min at 35 °C. Next, 4 μg/mL trypsin inhibitor (Soybean, Sigma, St. Louis, MO, USA) was added to stop the digestion. Both the un-nicked EL-HN (single chain, SC) and nicked EL-HN (dichain, DC) were determined by SDS-PAGE.

A recombinant plasmid pET32a-SNAP-25 encoding human SNAP-25 protein (aa 1-206) with his tag was constructed in our Lab. The SNAP-25 protein was expressed in BL21(DE3) cells and purified by affinity chromatography. The proteolytic activity of EL-HN was assayed in vitro using the SNAP-25 protein. The assay was performed in a final volume of 50 µL (50 mM Hepes, pH 7.5, 5 mM DTT, 10 mmol/L NaCl and 0.1% Tween 20), containing different concentrations of EL-HN (from 2.56 nmol to 1600 nmol) or BoNT/E (from 4.8 nmol to 600 nmol and from 2 μmol or 5 μmol substrate SNAP-25). The amounts of cleavage of SNAP-25 (2 or 5 μmol/L) by the EL-HN or BoNT/E were determined by incubating the samples at 37 °C for 30 min. The reactions were stopped by adding the 4× concentrated SDS-PAGE sample buffers. The extent of cleavage was then evaluated following electrophoresis on SDS-PAGE gels.

### 5.4. Neurotoxicity of EL-HN In Vivo

The neurotoxicity of the un-nicked and nicked EL-HN in mice following an intraperitoneal injection as a full neurotoxin was determined using an LD_50_ assay. Groups of four mice were used for each concentration, and the un-nicked and nicked EL-HN were diluted in sterile normal saline. The lowest amount of toxin that killed 50% of mice within four days was considered to be one minimal lethal dose (LD_50_) and was expressed as the number of LD_50_ units/mg of protein.

### 5.5. Immunization of Mice and Challenge with BoNT/E

We divided 6–8-week-old specific pathogen-free (SPF) female Balb/c mice into groups (*n* = 10 per group), with a PBS group serving as the control. Single antigen (10 μg) and combination antigens (5 μg each) were diluted with sterile PBS and formulated with 0.2% (*w*/*w*) aluminum hydroxide gel (Alhydrogel; Brenntag Biosector, Frederikssund, Denmark). The mice were vaccinated intramuscularly with 100 μL of subunit vaccine preparation. The injection protocol was a total of three immunizations with an interval of 3 weeks. Then, 100~200 μL blood from each mouse was collected at 18 days after two or three vaccinations. After 2 or 3 immunizations, the 10^2^, 10^3^, or 10^4^ LD_50_ (50% lethal doses) BoNT/E was used to challenge the mice, and the survival rate was calculated for one week.

A dose-dependent protective experiment was carried out on the recombinant antigens with a better protective effect. The female Balb/c mice were immunized with once or twice using serially diluted (4000 ng, 1000 ng, 250 ng, 62.5 ng, 15.6 ng, and 3.9 ng) antigen and the negative control; 100~200 μL blood from each mouse was collected at 18 days after one or two vaccinations. These immunized mice were challenged with 10^2^, 10^3^, 10^4^ LD_50_ of BoNT/E (Iwanai strain,10^7^ LD_50_/mg), which is assayed using botulinum antitoxin standard (from National Institutes of Food and Drug Control, Beijing, China), 3 weeks after the last vaccination. The survival rate was observed once per day for a week.

### 5.6. Determination of Humoral Immune Response after Immunization with Recombinant Functional Fragment Antigens

Humoral immune responses were measured using an enzyme-linked immunosorbent assay (ELISA). Each well of a 96-well enzyme-linked plate was coated overnight at 4 °C with 100 μL of recombinant antigen (2 µg/mL). The plates were incubated with 200 μL of 2% skim milk blocking solution at 37 °C for 2 h and then washed three times with PBST (PBS with 0.1% (*v*/*v*) Tween-20) for 3 times. Mouse serum samples were 4-fold serially diluted from 1:100 using a 2% skim milk blocking solution. The diluted samples (100 µL) were added to each well and incubated for another 90 min at 37 °C. After washing with PBST six times, the plates were added with 100 µL of 1:5000 diluted IgG-HRP per well and incubated for 30 min at 37 °C. The samples were washed with PBST, incubated for 10 min at 37 °C, followed by adding 50 µL of citrate buffer (pH 5.0) with 0.02% (*v*/*v*) hydrogen peroxide and 0.04% (*w*/*v*) o-phenylenediamine to each well. Then, 2 M H_2_SO_4_ at 50 µL per well was used to stop the reaction, and the absorbance was read at 492 nm on a microplate reader. Absorbance values at 492 nm (OD_492_) that were greater than 0.5 and ratios of the absorption value of the experimental group and the negative control group greater than 2.1 were regarded as positive results. Serum samples taken from individual mice in each group were measured separately, and the geometric mean titer (GMT) for each group was calculated using the average values.

### 5.7. Determination of the Titer of Neutralizing Antibodies in Mouse Serum

A BoNT/E neutralization assay [26] was used to measure the neutralizing potency of the mouse sera. Briefly, serum samples from immunized mice were serially diluted before being mixed with a BoNT/E standard concentration solution (100 LD_50_/mL, 1 mL). Dilution buffer (50 mM KH_2_PO_4_, 50 mM Na_2_HPO_4_, 1M NaCL, 1% gelatin, pH 6.5) was then used to adjust the total volume to 2.5 mL before incubation at room temperature for 1 h to fully react the neurotoxin and antibodies. Female KM mice weighing 15~18 g were then injected with the mixtures intraperitoneally using 500 µL per mouse (four mice per group, one test sample has eight different groups). Starting at 12 h after injection, the survival of the mice was observed for a week, once every day. The serum neutralizing antibody titer were calculated as international units per milliliter (IU/mL) relative to the World Health Organization (WHO) BoNT/E antitoxin levels.

### 5.8. Statistical Analysis

One-way analysis of variance (ANOVA) or using Student’s *t*-test was used to analyze differences in the antibody immune responses between the groups. To ascertain the statistical differences in survival between the treatment groups, Fisher’s exact test was used. Statistical significance was accepted at *p* values < 0.05 for the data from all tests. Probit analysis at the 95% confidence level was used to determine the effective dose protecting half of the mice (ED_50_) using IBM SPSS Statistics (version 19.0, IBM Corp., Armonk, NY, USA).

## Figures and Tables

**Table 1:** Basic information for the BoNT/E functional fragments used in the present study.

Functional Fragment	Amino Acid Sequence	Fragment Size	Protein Molecular Weight	Fragment Feature
EL	1–422	1263 bp	50 kDa	Light chain enzyme active region
EHN	423–840	1254 bp	50 kDa	Transmembrane region
EL-HN	2–840	2517 bp	100 kDa	Light chain enzyme Transmembrane region
EHc	841–1252	1236 bp	50 kDa	Receptor-binding region
EHc-N	841–1063	669 bp	27 kDa	C-terminal of receptor-binding region
EHc-C	1056–1252	591 bp	24 kDa	N-terminal of receptor-binding region

**Table 2:** Protective ability and neutralization antibody titer in mice immunized using different functional fragment antigens.

Vaccine (10 μg)				Number of Survivors ^a^			NA (IU/mL) ^c^	
	Two Immunizations			Three Immunizations			Two Immunizations	Three Immunizations
	10^2 b^	10^3 b^	10^4 b^	10^2 b^	10^3 b^	10^4 b^		
EHc-N	0 *	0	ND	0 *	ND	ND	<0.25	<0.25
EHc-C	6	0 *	ND	10	10	10	<0.25	1
EHc-N + EHc-C	2 ^&^	0 *	ND	10	8	ND	<0.25	0.25
EHc	10	10	10	10	10	10	4	32
EL-HN	10	10	10	10	10	10	29.3	136.6
EHc + EL-HN	10	10	10	10	10	10	12.8	120
EHc + EHN	10	10	10	10	10	10	1	16
EHN	5 ^#^	0 *	ND	10	7	ND	<0.25	0.25
EL	10	6	0 *	10	10	10	0.5	2.25
EL + EHN	10	10	10	10	10	10	0.5	8
PBS	0	ND	ND	ND	ND	ND	<0.25	<0.25

ND—not determined. ^a^ The 10^2^, 10^3^, or 10^4^ LD50 of BoNT/E was used to challenge mice (*n* = 10/group) at 3 weeks after two or three immunizations. Data show the number of mice that remained alive (survivors). ^b^ The challenge dose of BoNT/E. ^c^ We pooled the sera in each group of mice because of the limited amount of available sera; therefore, we only determined the average neutralizing antibody (NA) titer of each group. * *p* = 0.00001 < 0.001, ^#^ *p* = 0.033 < 0.05, and ^&^ *p* = 0.0007 < 0.01, compared with both EHc and EL-HN groups.

**Table 3:** Survival and sera neutralization antibody titer of mice after immunization with different dosages of EHc or/and EL-HN antigens.

EHc (ng) ^a^	NA (IU/mL) ^b^	Number of Survivors ^c^		EL-HN (ng) ^a^	NA (IU/mL) ^b^	Number of Survivors ^c^		EL-HN + EHc (ng) ^a^	NA (IU/mL) ^b^	Number of Survivors ^c^	
	2 ^d^	1 ^d^	2 ^d^		2 ^d^	1 ^d^	2 ^d^		2 ^d^	1 ^d^	2 ^d^
4000	1.5	6	10	4000	29.3	10	10	4000	12.8	8	10
1000	0.5	3	10	1000	14.6	10	10	1000	8.5	8	10
250	<0.25	0	6	250	4	6	10	250	2.0	2	10
62.5	<0.25	0	4	62.5	1	0	10	62.5	0.5	0	10
15.6	<0.25	0	2	15.6	<0.25	0	4	15.6	<0.25	0	2
3.9	<0.25	0	0	3.9	<0.25	0	0	3.9	<0.25	0	0

**^a^** Mice were vaccinated with EHc or/and EL-HN antigens range of 3.9–4000 ng. ^b^ We pooled the sera of each mouse group to determine the average titer of neutralizing antibodies (NA). **^c^** Different doses of BoNT/E were used to challenge Balb/C mice (10 mice/group) after one or two immunizations. The mice immunized once were challenged with 10^2^ LD_50_ of BoNT/E. The mice immunized twice were challenged with 10^3^ LD_50_ of BoNT/E. The table shows the number of mice that remained alive (survivors). **^d^** Number of vaccinations.

## Data Availability

The data presented in this study are available upon request from the corresponding author.
